# Identification of antimicrobial resistant bacteria isolated from *Hyalomma excavatum* and *Hyalomma dromedarii* infesting camels in Aljouf region, Saudi Arabia

**DOI:** 10.3389/fvets.2025.1634753

**Published:** 2025-10-02

**Authors:** Mashal M. Almutairi, Abdulaziz Alouffi, Eman M. Damra, Moureq Alotaibi, Nora S. Alkahtani, Waleed S. Al Salem, Alanoud T. Aljasham

**Affiliations:** ^1^Department of Pharmacology and Toxicology, College of Pharmacy, King Saud University, Riyadh, Saudi Arabia; ^2^King Abdulaziz City for Science and Technology, Riyadh, Saudi Arabia; ^3^Department of Agriculture, Ministry of Environment, Water, and Agriculture, Riyadh, Saudi Arabia; ^4^Department of Clinical Laboratory Sciences, College of Applied Medical Sciences, King Saud University, Riyadh, Saudi Arabia

**Keywords:** tick, *Hyalomma excavatum*, *Hyalomma dromedarii*, antimicrobial resistance, antimicrobial agent

## Abstract

Ticks and tick-borne pathogens are expanding their geographic ranges to novel suitable habitats. Together with the World Health Organization (WHO) and the United Nations (UN), Saudi Arabia’s government has joined efforts to prevent the development of tick-associated pathogens. Here, we investigated the prevalence and diversity of antimicrobial-resistant (AMR) bacteria in ticks parasitizing camels in Al-Jouf province. A total of 60 ticks were sampled and identified as *Hyalomma excavatum* (*n* = 41) and *Hyalomma dromedarii* (*n* = 19), infesting 11 camels. Altogether 70 bacterial isolates were isolated and subjected to Gram staining, followed by identification using the Vitek 2 compact system. Bacterial isolates consisted of 23 different bacterial species. 68.6% (*n* = 48) of the total isolates were identified as Gram-positive bacteria, comprising 14 different species, while 31.4% (*n* = 22) of the total isolates were Gram-negative bacteria, comprising 9 different species. Each collected tick was found positive for at least one bacterial species, however, 9 out of the 70 ticks were found to carry 2 or 3 bacterial species. Antimicrobial susceptibility testings showed that the isolated bacteria exhibited resistance to several clinical antimicrobial agents. The antimicrobial susceptibility profile of Gram-positive bacteria showed that 100% (*n* = 30) were resistant to benzylpenicillin; 93.3% (*n* = 28) were resistant to and oxacillin; 56.7% (*n* = 17) were resistant to clindamycin; 53.3% (*n* = 16) were resistant to vancomycin; 43.3% (*n* = 13) were resistant to rifampicin; 40% (*n* = 12) were resistant to erythromycin and trimethoprim/sulfamethoxazole; 30% (*n* = 9) were resistant to teicoplanin; 3.3% (*n* = 1) was resistant to tetracycline. All Gram-positive bacteria were 100% susceptible to linezolid, gentamicin, tobramycin, levofloxacin, moxifloxacin, and tigecycline. Susceptibility testing for Gram-negative bacteria revealed, 75% (*n* = 12) were resistant to cefoxitin, whereas 68.75% (*n* = 11) were resistant to ampicillin. 62.5% (*n* = 10) of the Gram-negative bacteria were resistant to ceftazidime. In addition, 50% (*n* = 8) were resistant to cephalothin, ceftriaxone, and trimethoprim/sulfamethoxazole; 43.75% (*n* = 7) were resistant to cefepime; 31.25% (*n* = 5) were resistant to amoxicillin/clavulanic acid; 6.25% (*n* = 1) was resistant to nitrofurantoin. However, all Gram-negative bacteria were susceptible to other antimicrobials including piperacillin/ tazobactam, imipenem, meropenem, amikacin, gentamicin, ciprofloxacin and tigecycline. The current study sheds light on the AMR burden in ticks infesting camels in Al-Jouf province.

## Introduction

Ticks are hematophagous ectoparasites that have significant effects on both human and animal health due to the pathogens they carry (1, 2). They are not only vectors for known pathogens such as Borrelia, Anaplasma, and Rickettsia, but they also harbor a diverse community of commensal bacteria that include endosymbionts, skin-associated organisms, opportunists, and pathogens acquired from blood meals, the host skin, and from the environment. Community composition can differ by host, geography, sex, and season (3, 4).

When ticks bite, they can transfer disease-causing microorganisms into the bloodstream of their hosts, leading to various illnesses (5, 6). In addition to the health consequences, ticks also have a significant economic impact (7, 8). The treatment and management costs associated with these illnesses can be substantial, including expenses for medical care, diagnostic tests, and long-term healthcare impacts. Furthermore, ticks can have indirect economic effects, such as reduced productivity in affected individuals and the need for preventive measures in high-risk areas (7, 9).

In this study, tick-associated pathogens refer to bacterial species or genera with documented pathogenic potential that are cultured or isolated from ticks. This indicates association, not proven transmission. For example, opportunistic pathogens such as *Staphylococcus aureus* and *Escherichia coli* have been recovered from field ticks, including *Hyalomma dromedarii* collected from camels (1, 10). By contrast, tick-borne disease (TBD) refers to clinical illness in animals or humans caused by a pathogen that has been proven to be transmitted by ticks—for example, *Theileria annulata* with *Hyalomma* ticks, and, beyond *Hyalomma* systems, *Anaplasma phagocytophilum* (human granulocytic anaplasmosis) and *Borrelia burgdorferi* (Lyme disease) transmitted by *Ixodes* ticks (5, 11–13). Accordingly, we report antimicrobial susceptibility profiles of tick-associated pathogens isolates and do not infer tick-borne transmission unless such evidence exists.

Recent studys showed that ticks can carry antimicrobial-resistance (AMR) bacteria and genes (ARGs), often among non-pathogenic or opportunistic bacteria, indicating potential exchange of resistance determinants at the tick–host–environment interface (1, 14, 15). At the same time, several classical tick-borne pathogens appear to harbor relatively few ARGs, which supports profiling both resistomes and phenotypic susceptibility of cultured isolates (16). Within this One Health context, our culture-based AMR data from *Hyalomma* ticks on camels complement sequence-based studies and provide actionable information for veterinary care and surveillance (17).

*H. dromedarii* is the predominant camel tick across arid and semi-arid regions of the Middle East and North Africa and is relevant to zoonotic risk in these settings; *H. excavatum* has been reported as a vector or putative vector of several pathogens affecting livestock (5, 13, 18). By characterizing which culturable bacteria are present in *H. dromedarii* and *H. excavatum* on camels and how resistant they are to clinically relevant agents, our results will inform veterinary care, stewardship, and One Health surveillance in settings where animal–human–environment interfaces are close (2, 17). Given the region’s economic and cultural value of camels and the new global focus on antimicrobial resistance, the implications of these results lean toward the ticks as being the unsung disseminators of resistant bacteria (19, 20). These data can be used to guide veterinary and public health surveillance activities, facilitate the development of targeted control measures, and establish national capacities in alignment with WHO’s One Health approach to combat antimicrobial resistance (17, 21).

This study aims to identify and characterize bacterial species isolated from *Hyalomma excavatum* and *Hyalomma dromedarii* ticks infesting camels in Al-Jouf province, Saudi Arabia, to evaluate the antimicrobial susceptibility profiles of the isolated bacteria using standardized laboratory methods and to assess the prevalence of antimicrobial resistance, including multidrug resistance, among the detected bacterial species and to highlight their potential impact on animal and public health. Understanding and addressing AMR in ticks is crucial for protecting both animal and public health.

## Materials and methods

### Collection and identification of ticks

In August 2023, 60 ticks were collected from 11 female dromedary camels (*Camelus dromedaries*) at the camel market in Al-Jouf province, Saudi Arabia (29.8874° N and 39.3206° E) (Supplementary Table 1). Al-Jouf area has been chosen for many reasons, as discussed in the previous study (1). The ticks were stored in a jar containing 70% ethanol and transported to the College of pharmacy, King Saud University for further testing. Taxonomic identification of collected specimens was conducted by examining the morphological characters of the capitulum, scutum, and idiosoma to the species level, together with recording of the developmental stage (larva, nymph, adult) and sex (male, female) using morphological keys (Leica EZ4HD stereomicroscope) (22, 23). The study involved the manual collection of ticks from camels using fine-tipped forceps, a widely accepted, non-invasive veterinary practice commonly employed for tick removal in animals. No invasive procedures or experimental manipulations were conducted on the camels, and the collection process did not cause distress or harm to the animals. Consequently, formal ethical approval was not required, in accordance with institutional and international guidelines. All methods performed complied strictly with relevant veterinary standards and local regulations.

All owners of the 11 female dromedary camels were verbally informed, and permission was taken from them.

### Isolation of bacteria from ticks

Following identification, ticks were washed with 70% ethanol and rinsed 3 times with phosphate-buffered saline (PBS). Sterile forceps and blades were used to remove the ticks’ cuticle, and their internal organs were then dissected and placed in tubes. Ticks were individually homogenized (whole-tick) with PBS using an electric homogenizer (MSE Supplies LLC, US) to culture associated bacteria and obtain phenotypic antimicrobial-susceptibility data from the full organismal compartment. 15 mL tubes of nutrient broth media were incoluated with homogenates and incubated for 24 h at 37 °C on a shaker (250 rpm) (10, 24). Various media were used to plate the growing cultures, including blood and MacConkey agar to allow a wide range of bacteria to grow (25). Approximately 1 to 2 colonies from each plate were selected based on their morphology (color, structure, shape, and size) after 24 h of incubation at 37 °C. Upon isolation, the bacteria were stored with glycerol at −80 °C until further analysis.

### Identification of bacteria

Following Gram staining, bacteria were identified using the Vitek 2 compact system (bioMérieux Inc. USA) (26). Gram-positive and Gram-negative specimens were identified using GP ID REF21342 cards and GN ID REF21341 cards, respectively, as per manufacturer’s instructions.

### Antimicrobail agents susceptibility

A total of 70 bacterial isolates were obtained from collected ticks, of which 46 underwent antimicrobial susceptibility testing using the Vitek 2 compact system (bioMérieux Inc. USA). The susceptibilities of the remaining 24 bacterial isolates were not tested due to a lack of Vitek cards appropriate for these isolates. The minimum inhibitory concentration (MIC) of antimicrobial agents for each pathogen was determined using the AST-P580 card (for *Staphylococcus* spp., *Enterococcus* spp., and *Streptococcus agalactiae*) and the AST-N291 card (for Gram-negative bacilli) cards (bioMérieux Inc. USA). Antimicrobial classes tested include: penicillins (ampicillin, amoxicillin/clavulanic acid, piperacillin/tazobactam, benzylpenicillin, oxacillin); aminoglycosides (gentamicin, tobramycin, amikacin); cephalosporin (cephalothin, cefoxitin, ceftazidime, ceftriaxone, cefepime); carbapenem (imipenem, meropenem); fluoroquinolone (ciprofloxacin, levofloxacin, moxifloxacin), tetracyclines (tetracycline, tigecycline); glycopeptide (teicoplanin, vancomycin); macrolides (erythromycin); lincomycin (clindamycin); oxazolidinone (linezolid); rifamycin (rifampicin); nitrofuran (nitrofurantoin); Sulfonamides (trimethoprim/sulfamethoxazole). These agents were tested against Gram-positive or Gram-negative bacteria based on their known or expected primary activity. For all tests, the following quality control strains were used: *E. coli* ATCC 25922 and 35218, *Staphylococcus aureus* ATCC 29213, *Pseudomonas aeruginosa* ATCC 27853, *Enterococcus faecalis* ATCC 29212, *Haemophilus influenzae* ATCC 49247 and 49766, and *Streptococcus pneumoniae* ATCC 49619. As per the guidelines of the National Committee for Clinical Laboratory Standards (NCCLS), USA, MIC cutoff values were used to distinguish sensitive, intermediate, and resistant bacteria. The results were analyzed using the Vitek 2 compact software version 07.01.

### Statistical analysis

Data were summarized at 2 levels: tick level (number and percentage of *H. excavatum H. dromedarii* collected; sex distribution; number of ticks yielding at least one isolate) and isolate level (species distribution and antimicrobial susceptibility). Antimicrobial susceptibility testing (AST) results from the VITEK 2 system were classified as susceptible (S), intermediate (I), or resistant (R) according to CLSI interpretive criteria as implemented in VITEK 2. For each antimicrobial agent, counts (n) and percentages (%) of S, I, and R were calculated using, as the denominator, the number of isolates actually tested for that agent; isolates without an AST result for a given drug were excluded from that drug’s denominator.

Multidrug resistance (MDR) was defined *a priori* as resistance (R) to at least one agent in 3 or more antimicrobial classes; intermediate (I) results were not counted as resistance for MDR classification. MDR proportions were reported overall and, where relevant, by bacterial species.

Because this was a baseline, culture-based survey with a modest sample size and no prespecified group comparisons, no formal hypothesis testing was performed. Results are presented as descriptive statistics (frequencies and percentages). Data collation and tabulations were performed using standard spreadsheet/statistical software, and tables/figures summarize S/I/R patterns in line with these summaries.

## Results

### Identification of the isolated bacteria

**Figure 1:**
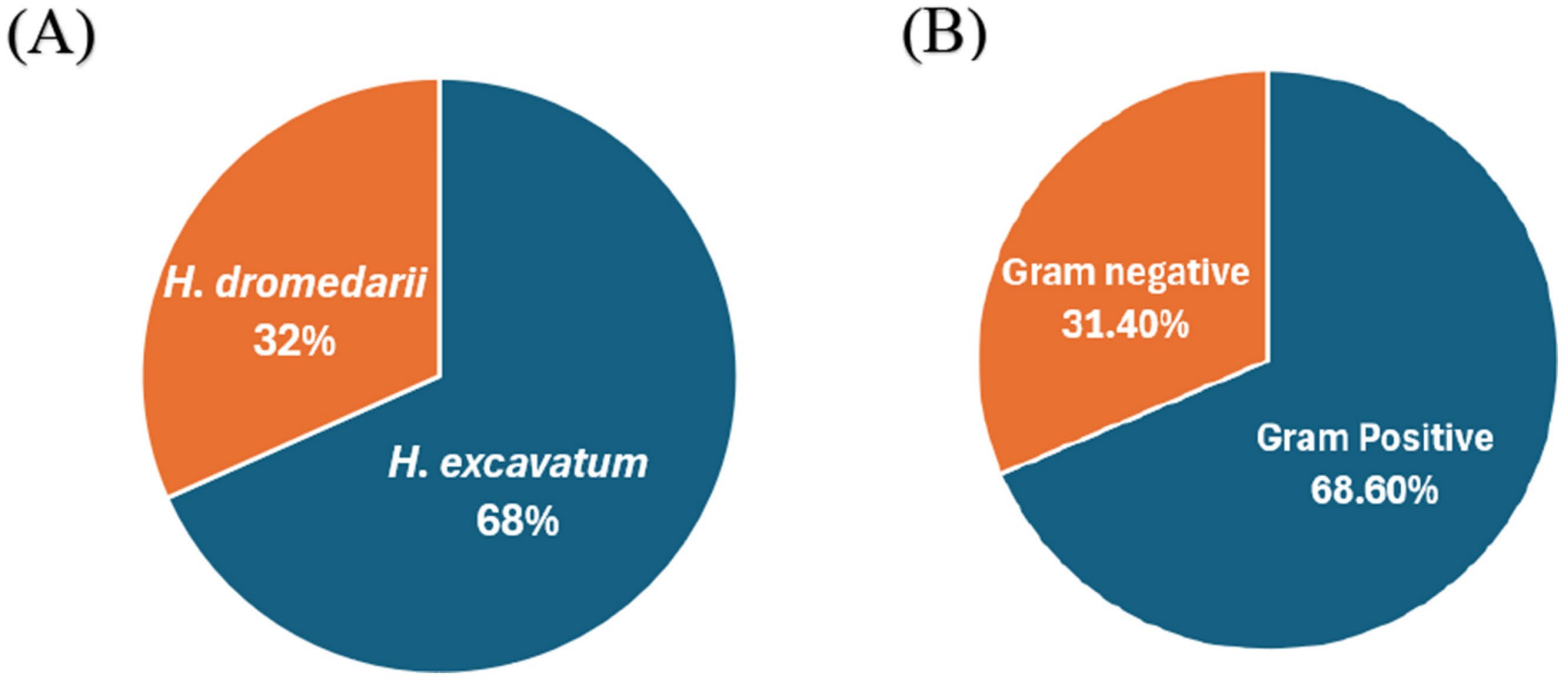
The percentages of the tick species and bacterial types used in the study. **(A)** The distributaion of *H. dromedarii* and *H. excavatum*, **(B)** the percentages of Gram-positive and Gram-negative bacteria from the collected ticks.

Sixty ticks were collected from 11 female dromedary camels and classified as: adult *H. excavatum* (68.3% *n* = 41), of which 32 (78%) were females and 9 (22%) were males, and adult *H. dromedarii* (31.7% *n* = 19), of which 9 (47.4%) were females and 10 (52.6%) were males (Figure 1A). A total of 70 bacteria were isolated. 68.6% (*n* = 48) of the total isolates were identified as Gram-positive bacteria, and 31.4% (*n* = 22) were identified as Gram-negative bacteria (Figure 1B). Gram positive bacteria compromised of 14 different species: *Aerococcus viridans* (*n* = 12), *Staphylococcus lentus* (*n* = 11), *Staphylococcus pseudintermedius* (*n* = 7), *Staphylococcus haemolyticus* (*n* = 4), *Staphylococcus sciuri* (*n* = 4), *Enterococcus casseliflavus* (*n* = 2), *Staphylococcus aureus* (*n* = 1), *Staphylococcus vitulinus* (*n* = 1), *Staphylococcus hominis* (*n* = 1), and *Streptococcus equi ssp zooepidemicus* (*n* = 1), *Mic.luteus lylae* (*n* = 1), *Gemella morbillorum* (*n* = 1), *Kocuria* var*ians* (*n* = 1) and *Granulicatella elegans* (*n* = 1). Gram-negative bacteria compromised of 9 different species: *Sphingomonas paucimobilis* (*n* = 13), *Gardnerella vaginalis* (*n* = 2), *Pantoea* spp. (*n* = 1), *Acinetobacter baumannii* (*n* = 1), *Stenotrophomonas maltophilia* (*n* = 1), *Vibrio vulnificus* (*n* = 1), *Cronobacter sakazakii group* (*n* = 1), *Neisseria animaloris* (*n* = 1), *and Methylobacterium* spp. (*n* = 1). Every tick was found to be positive for at least one bacterial species. However, 9 out of 60 ticks contained 2 or 3 bacterial species (Supplementary Table 1).

### Antimicrobial agents susceptibility

A total of 46 bacterial isolates (30 Gram-positive and 16 Gram-negative isolates) were tested for antimicrobial susceptibility using a Vitek 2 compact system. The results indicated that both Gram-positive and Gram-negative bacteria are resistant to a number of antimicrobial agents (Table 1). The antimicrobial susceptibility profile of Gram-positive bacteria showed that 100% (*n* = 30) were resistant to benzylpenicillin; 93.3% (*n* = 28) were resistant to oxacillin; 56.7% (*n* = 17) were resistant to clindamycin; 53.3% (*n* = 16) were resistant to vancomycin; 43.3% (*n* = 13) were resistant to rifampicin; 40% (*n* = 12) were resistant to erythromycin and trimethoprim/sulfamethoxazole; 30% (*n* = 9) were resistant to teicoplanin; 3.3% (*n* = 1) was resistant to tetracycline (Table 1). All Gram-positive bacteria were 100% susceptible to linezolid, gentamicin, tobramycin, levofloxacin, moxifloxacin, nitrofurantoin and tigecycline (Table 1).

**Table 1:** Antimicrobial susceptibility of Gram-positive and Gram-negative bacteria isolated from *H. excavatum* and *H. dromedarii*.

Gram-positive bacteria																
Pattern	Benzylpenicillin	Oxacillin	Gentamicin	Tobramycin	Levofloxacin	Moxifloxacin	Erythromycin	Clindamycin	Linezolid	Teicoplanin	Vancomycin	Tetracycline	Tigecycline	Nitrofurantoin	Rifampicin	Trimethoprim/Sulfamethoxazole
S	0	6.7% (2)	100% (30)	100% (30)	100% (30)	100% (30)	26.7% (8)	40% (12)	100% (30)	63.3% (19)	46.7% (14)	83.4% (25)	100% (30)	86.7% (26)	50% (15)	60% (18)
I	0	0	0	0	0	0	33.3% (10)	3.3% (1)	0	6.7% (2)	0	13.3% (4)	0	13.3% (4)	6.7% (2)	0
R	100% (30)	93.3% (28)	0	0	0	0	40% (12)	56.7% (17)	0	30% (9)	53.3% (16)	3.3% (1)	0	0	43.3% (13)	40% (12)

Gram-negative bacteria																
Pattern	Ampicillin	Amoxicillin/Clavulanic acid	Piperacillin/Tazobactam	Cefalotin	Cefoxitin	Ceftazidime	Ceftriaxone	Cefepime	Imipenem	Meropenem	Amikacin	Gentamicin	Ciprofloxacin	Tigecycline	Nitrofurantoin	Trimethoprim/Sulfamethoxazole
S	25% (4)	50% (8)	100% (16)	43.75% (7)	25% (4)	31.25% (5)	37.5% (6)	37.5% (6)	100% (16)	100% (16)	100% (16)	100% (16)	100% (16)	100% (16)	75% (12)	50% (8)
I	6.25% (1)	18.75% (3)	0	6.25% (1)	0	6.25% (1)	12.5% (2)	18.75% (3)	0	0	0	0	0	0	18.75% (3)	0
R	68.75% (11)	31.25% (5)	0	50% (8)	75% (12)	62.5% (10)	50% (8)	43.75% (7)	0	0	0	0	0	0	6.25% (1)	50% (8)

S, susceptible; I, intermediate resistance; R, resistant. The numbers in the parentheses indicate the number of bacteria.

Regarding Gram-negative bacteria, 75% (*n* = 12) showed resistance to cefoxitin, while 68.75% (11) demonstrated resistance to ampicillin. A total of 62.5% (*n* = 10) of the Gram-negative bacteria were resistant to ceftazidime. Furthermore, 50% (*n* = 8) exhibited resistance to cephalothin, ceftriaxone, and trimethoprim/sulfamethoxazole; 43.75% (*n* = 7) demonstrated resistance to cefepime; 31.25% (*n* = 5) displayed resistance to amoxicillin/clavulanic acid; 6.25% (*n* = 1) displayed resistance to nitrofurantoin. Nevertheless, all Gram-negative bacteria were susceptible to other antimicrobials including piperacillin/tazobactam, imipenem, meropenem, amikacin, gentamicin, and ciprofloxacin and tigecycline (Table 1).

**Figure 2:**
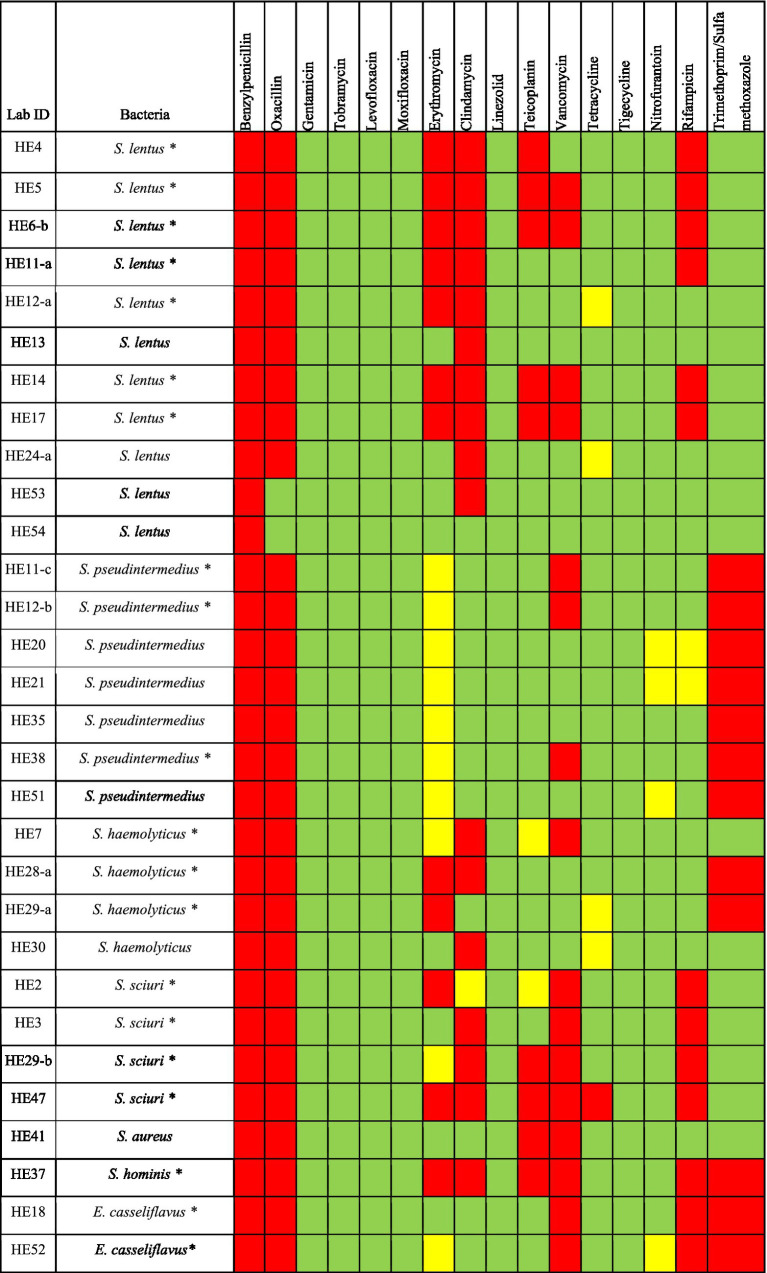
Antimicrobial susceptibility of Gram-positive bacteria isolated from *H. excavatum* and *H. dromedarii*. Green, susceptible; Yellow, intermediate resistance; Red, resistant. *Denotes MDR bacteria.

All the Gram-positive bacteria tested for antimicrobial agents susceptibility showed resistance to 1 or more classes of antimicrobials. Among *S. lentus* isolates (*n* = 11), all isolates showed resistance to benzylpenicillin; 10 isolates exhibited resistance to clindamycin; 9 isolates showed resistance to oxacillin; 7 isolates were resistant to erythromycin; 6 isolates showed resistance to rifampicin; 5 isolates displayed resistance to teicoplanin; 4 isolates were resistant to vancomycin. All *S. pseudintermedius* isolates (*n* = 7) showed resistance to benzylpenicillin, oxacillin and trimethoprim/sulfamethoxazole, and 3 isolates exhibited resistance to vancomycin. For *S. haemolyticus* (*n* = 4), all isolates were resistant to benzylpenicillin and oxacillin, 2 isolates showed resistance to erythromycin, clindamycin and trimethoprim/sulfamethoxazole. All *S. sciuri* (*n* = 4) isolates demonstrated resistance to benzylpenicillin, oxacillin, vancomycin and rifampicin; 3 isolates showed resistance to clindamycin; 2 isolates were resistant to erythromycin and teicoplanin; 1 isolate was resistant to tetracycline. Both *E. casseliflavus* (*n* = 2) isolates were resistant to benzylpenicillin, oxacillin, vancomycin, rifampicin and trimethoprim/sulfamethoxazole. The *S. aureus* isolate showed resistance to benzylpenicillin, oxacillin, teicoplanin and vancomycin. The *S. hominis* isolate showed resistance to benzylpenicillin, oxacillin, erythromycin, clindamycin teicoplanin and vancomycin. The *E. casseliflavu* isolate showed resistance to benzylpenicillin, oxacillin, vancomycin, rifampicin and trimethoprim/sulfamethoxazole (Figure 2).

**Figure 3:**
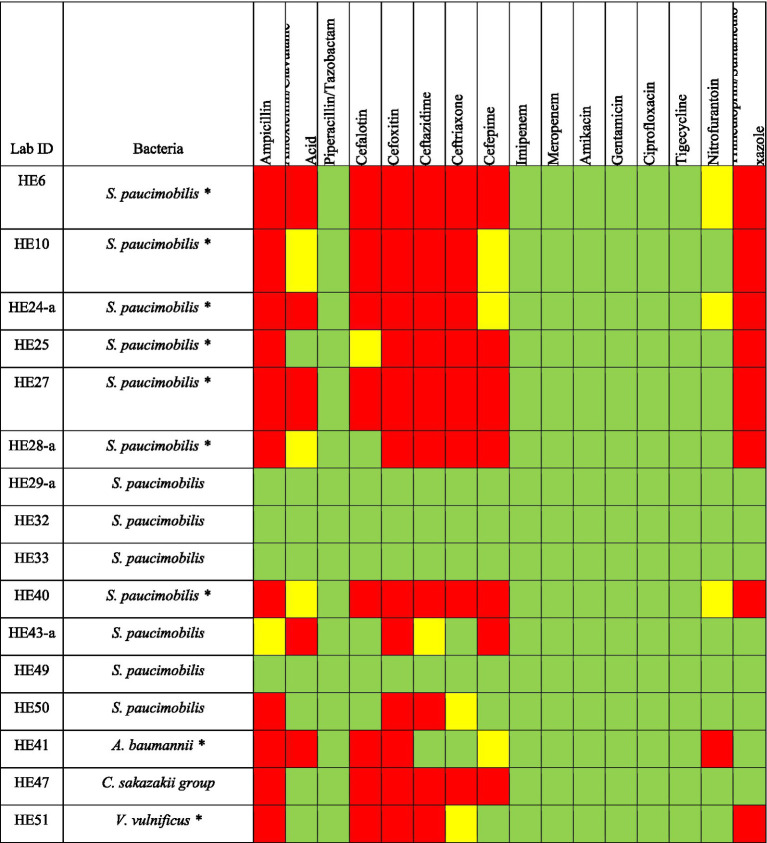
Antimicrobial susceptibility of Gram-negative bacteria isolated from *H. excavatum* and *H. dromedarii*. Green, susceptible; Yellow, intermediate resistance; Red, resistant. *Denotes MDR bacteria.

Among the Gram-negative bacteria tested for antimicrobial susceptibility, 12 out of 16 isolates showed resistance to 1 or more classes of antimicrobial. For *S. paucimobilis* (*n* = 13), 8 isolates showed resistance to ampicillin and ceftazidime; 7 isolates showed resistance to ceftriaxone; and trimethoprim/sulfamethoxazole; 6 isolates showed resistance to cefepime; 5 isolates showed resistance to cephalothin; 4 isolates showed resistance to amoxicillin/clavulanic; 9 isolates showed resistance to cefoxitin. *A. baumannii* exhibited resistance to ampicillin, amoxicillin/clavulanic, cephalotin, cefoxitin and nitrofurantoin. The *C. sakazakii* group elicited resistance to ampicillin, cefalotin, cefoxitin, ceftazidime, ceftriaxone and cefepime. *V. vulnificus* was resistant to ampicillin, cephalotin, cefoxitin, ceftazidime and trimethoprim/sulfamethoxazole (Figure 3).

In order to identify multidrug resistant (MDR) bacteria, the isolates that were resistant to 3 or more antimicrobial agents were examined. Results revealed that 66.7% (*n* = 20) of Gram-positive bacteria were MDR bacteria, with some isolates being resistant to more than 4 classes of antimicrobials (Figure 2). Of the Gram-positive bacteria species, MDR was identified in: *S. lentus* (63.6%, *n* = 7/11); *S. pseudintermedius* (42.8%, *n* = 3/7); *S. haemolyticus* (75%, *n* = 3/4); *S. sciuri* (100%, *n* = 4/4); *S. hominis* (100%, *n* = 1/1); *E. casseliflavu* (100%, *n* = 2/2) (Figure 2).

56.25% (*n* = 9) of Gram-negative bacteria were MDR bacteria, with some isolates again being resistant to more than 4 antimicrobial classes (Figure 3). A total of 7 isolates of *S. paucimobilis* (*n* = 7/13), *A. baumannii* and *V. vulnificus* showed resistance to 3 or more different classes af antimicrobial (Figure 3).

## Discussion

Ticks are obligate hematophagous ectoparasites that play an important role in the transmission of many pathogens to various hosts (27). Ticks are highly prevalent in Saudi Arabia, and hinder the development of farm animal production. The study is a baseline, culture-based study of bacteria associated with *H. excavatum* and *H. dromedarii* infesting camels. We focused on viable isolates to generate phenotypic antimicrobial-susceptibility profiles, which provide actionable information for clinical stewardship and complement sequence-based studies of tick microbiomes (e.g., 16S or metagenomics) (3, 4, 28).

Studies of *H. dromedarii* and *H. excavatum* show mixed communities including endosymbionts plus skin- and environment-associated organisms; community composition varies by host, geography, sex, and season (28–31). Regional culture-based work and other studies frequently recover *staphylococci* and related Gram-positive bacteria from *Hyalomma*, alongside environmental genera (1, 10, 20, 24). Our results align with these observations: Gram-positive organisms were common, and we isolated *Sphingomonas* and other environmental/opportunistic genera (32). The antimicrobial-resistance profiles observed here add phenotypic evidence to the sequencing literature and help prioritize surveillance and stewardship actions in camel production systems.

In line with other studies and likely due to their prevalence as commensal on ticks (24, 33), Gram-positive bacteria were the most common isolates with a prevalence of 66.7% (*n* = 40). For example, *Staphylococcus* spp. considered the most prevalent Gram-positive isolates in our study, in accordance with previous studies carried out in Saudi Arabia and Iraq, where *S. lentus* and *S. aureus* were frequently isolated from camel and cattle ticks (1, 10, 24). The increased prevalence of Gram-positive cocci may be due to contamination of the camel or tick from normal human skin flora as a result of animal-human contact. The prevalence of Gram-negative bacterial isolates in the current study was 33.3% (*n* = 20), which is in line with the findings of a previous study (20)*. S. paucimobilis* was the predominant Gram-negative bacteria and, to our knowledge, has not previously been reported in any tick species. Such a high detection rate of *S. paucimobilis* isolates might be due to the wider prevalence of this organism in the environment (drinking water, soil and plants) (32, 34–36). The extensive soil and water contamination among camel farms might assist in increasing infection of ticks with this bacteria.

Sequence- and culture-based studies show that ixodid ticks, including *Hyalomma*, harbor mixed communities of endosymbionts, skin-associated taxa, and environmental opportunists, influenced by host blood meals and ecological exposures (3, 4). Regionally, *H. dromedarii* on camels has yielded diverse culturable bacteria in prior work from Al-Jouf (1) and other surveys of ticks report frequent recovery of staphylococci and related Gram-positive genera (10, 24). Our results align with this pattern: *staphylococci* dominated among Gram-positive isolates, and environment-linked genera (e.g., *Sphingomonas*) were common among Gram-negatives, consistent with acquisition from skin and surroundings during host contact and off-host stages (20, 32). Notably, the AMR and multidrug-resistance profiles we observed fit broader evidence that resistant phenotypes can be enriched among opportunistic tick-associated bacteria, underscoring their relevance for veterinary care and one health surveillance (37, 38).

About 18 bacterial species in our isolated ticks were identified. The findings are replicated elsewhere (1), however tick-borne pathogens vary by region, time (19) and ecological conditions (39, 40). Differing bacterial species have been isolated not only from adult ticks, but also from their eggs, larvae and nymphs (40, 41). Indeed, these life stages increase the probability of bacterial transmission and the harboring of many different bacterial species by ticks reflects the variations in ecological environments (42). Ticks may acquire infections from the environment (4) or from Infected Hosts blood meals (3).

The research contributes to the preliminary understanding of AMR bacteria in ticks by providing data on the prevalence of AMR among bacterial isolates taken from ticks infesting camels in Al-Jouf province. Overall, the prevalence of AMR among Gram-positive bacteria was 100 and 75% in the Gram-negative bacteria. Additionally, a high prevalence of MDR was observed among Gram-positive bacteria 77% (*n* = 20), *S. lentus* (77.7%, *n* = 7/9) and *S. pseudintermedius* (50%, *n* = 3/6) showed a particularly high MDR profile. Among Gram-negative bacteria, 56.25% (*n* = 9) were MDR in the present study. Overall, 48.3% of the culture-confirmed bacterial isolates were MDR. The observed MDR pattern of the isolated bacteria might be linked to over-prescription of broad-spectrum antibiotics, lack of regular screening of antimicrobial resistance patterns before prescription, self-medication practice, or the misuse of antibiotics (43). The AMR pattern of bacteria in ticks might vary by place and time due to differences in drug regulatory policies, bacterial ecology (21, 38),bacterial strains, laboratory facilities and procedures, bacterial load, and community awareness of drug resistance (37).

Additionally, since we use complete tissue homogeneous, our data do not resolve the specific localization of the tissue of microbes. The work at the organ level is important because the physiology of the medium midgut and the immune defense shape the bacteria that persist after blood meal, and only a small number of pathogens usually pass through the midgut and spread via the hemolymph to the salivary glands or ovaries, positioning them for transmission in subsequent feedings (44, 45). Future research should combine organ-specific dissection with cultural-independent methods (e.g., 16S/shotgun genomes, FISH, qPCR) to map the location and better estimate the transmission potential (46).

Although many taxa we cultured are considered environmental or skin opportunists rather than classical tick-borne pathogens, they are epidemiologically relevant for 3 reasons. First, ticks sit at the animal–human–environment interface and can harbor antimicrobial-resistance genes (ARGs) that are frequently enriched in non-pathogenic/opportunistic taxa; these ARGs can circulate across hosts and settings (14, 15). Second, opportunists with phenotypic resistance can still cause disease in livestock (e.g., wound/mastitis or secondary infections) and pose occupational risks for handlers and abattoir workers, complicating empiric therapy and stewardship (17). Third, blood-meal inputs and environmental exposures can periodically reseed ticks with ARG-bearing bacteria (3), creating opportunities for horizontal gene transfer to co-resident microbes under antibiotic selection pressures (15). In this One Health context, culture-based susceptibility profiles of opportunists complement sequence-based microbiome surveys and provide actionable data for veterinary care and surveillance, especially since several canonical tick-borne pathogens appear to carry few ARGs (16).

## Conclusion

The prevalence of AMR bacteria from ticks parasitizing camels was significantly high in the study area. Strict guidelines and drug regulation policies should be in place for the prevention and control of AMR. Additionally, bacterial isolation and antimicrobial susceptibility testing of ticktik infested livestock should be routinely performed, and public health measures are pivotal to tackling diseases caused by ticks’ transmitted pathogens.

## Data Availability

The original contributions presented in the study are included in the article/Supplementary material, further inquiries can be directed to the corresponding authors.
