# Supplementary material for: Identification of antimicrobial resistant bacteria isolated from Hyalomma excavatum and Hyalomma dromedarii infesting camels in Aljouf region, Saudi Arabia
Source: Front Vet Sci. 2025 Oct 2;12:1634753. doi: 10.3389/fvets.2025.1634753 (PMC12527837; doi:10.3389/fvets.2025.1634753)
Supplement: Supplementary file 1 [file Table_1.docx]

**Supplementary Table 1: Information about the number of *Hyalomma excavatum and Hyalomma dromedarii* ticks that have been collected from different camels and the bacterial isolates from each tick.**

| **Tick number** | **Tick specie** | **Sex Of Ticks** | **Isolated Bacteria** | Lab ID |
| --- | --- | --- | --- | --- |
| **1** | *H. excavatum* | Female | *A. viridans* | HE1 |
| **2** | *H. excavatum* | Female | *S. sciuri* | HE2 |
| **3** | *H. excavatum* | Male | *S. sciuri* | HE3 |
| **4** | *H. excavatum* | Female | *S. lentus* | HE4 |
| **5** | *H. excavatum* | Female | *S. lentus* | HE5 |
| **6** | *H. excavatum* | Female | *S. paucimobilis* | HE6-a |
|  |  |  | *S. lentus* | HE6-b |
| **7** | *H. excavatum* | Female | *S. haemolyticus* | HE7 |
| **8** | *H. excavatum* | Female | *A. viridans* | HE8 |
| **9** | *H. excavatum* | Female | *S. paucimobilis* | HE9 |
| **10** | *H. excavatum* | Female | *S. equi ssp zooepidemicus* | HE10 |
| **11** | *H. excavatum* | Female | *S. lentus* | HE11-a |
|  |  |  | *A. viridans* | HE11-b |
|  |  |  | *S. pseudintermedius* | HE11-c |
| **12** | *H. excavatum* | Female | *S. lentus* | HE12-a |
|  |  |  | *S. pseudintermedius* | HE12-b |
| **13** | *H. excavatum* | Male | *S. lentus* | HE13 |
| **14** | *H. excavatum* | Male | *S. lentus* | HE14 |
| **15** | *H. excavatum* | Male | *S. Vitulinus* | HE15 |
| **16** | *H. excavatum* | Male | *A. viridans* | HE16 |
| **17** | *H. excavatum* | Male | *S. lentus* | HE17 |
| **18** | *H. excavatum* | Female | *E. casseliflavus* | HE18 |
| **19** | *H. excavatum* | Female | *A. viridans* | HE19 |
| **20** | *H. excavatum* | Female | *S. pseudintermedius* | HE20 |
| **21** | *H. excavatum* | Male | *S. pseudintermedius* | HE21 |
| **22** | *H. excavatum* | Female | *A. viridans* | HE22 |
| **23** | *H. excavatum* | Male | *S. paucimobilis* | HE23 |
| **24** | *H. excavatum* | Female | *S. lentus* | HE24-a |
|  |  |  | *S. paucimobilis* | HE24-b |
| **25** | *H. excavatum* | Male | *A. viridans* | HE25 |
| **26** | *H. excavatum* | Female | *S. paucimobilis* | HE26 |
| **27** | *H. excavatum* | Female | *S. paucimobilis* | HE27 |
| **28** | *H. excavatum* | Female | *S. haemolyticus* | HE28-a |
|  |  |  | *S. paucimobilis* | HE28-b |
| **29** | *H. excavatum* | Female | *S. haemolyticus* | HE29-a |
|  |  |  | *S. sciuri* | HE29-b |
| **30** | *H. excavatum* | Female | *S. haemolyticus* | HE30 |
| **31** | *H. excavatum* | Female | *A. viridans* | HE31-a |
|  |  |  | *S. paucimobilis* | HE31-b |
| **32** | *H. excavatum* | Female | *S. paucimobilis* | HE32 |
| **33** | *H. excavatum* | Female | *G. morbillorum* | HE33 |
| **34** | *H. excavatum* | Female | *A. viridans* | HE34 |
| **35** | *H. excavatum* | Female | *S. pseudintermedius* | HE35 |
| **36** | *H. excavatum* | Female | *Pantoea spp* | HE36 |
| **37** | *H. excavatum* | Female | *S. hominis ssp hominis* | HE37 |
| **38** | *H. excavatum* | Female | *S. pseudintermedius* | HE38 |
| **39** | *H. excavatum* | Female | *S. paucimobilis* | HE39 |
| **40** | *H. excavatum* | Female | *A. baumannii* | HE40 |
| **41** | *H. excavatum* | Female | *S. aureus* | HE41 |
| **42** | *H. dromedarii* | Female | *S. paucimobilis* | HE42 |
| **43** | *H. dromedarii* | Female | *A. viridans* | HE43-a |
|  |  |  | *G. vaginalis (variable staining)* | HE43-b |
| **44** | *H. dromedarii* | Female | *S. maltophilia* | HE44 |
| **45** | *H. dromedarii* | Female | *G. vaginalis (variable staining)* | HE45 |
| **46** | *H. dromedarii* | Female | *M. luteus lylae* | HE46-a |
|  |  |  | *C. sakazakii group* | HE46-b |
| **47** | *H. dromedarii* | Female | *S. sciuri* | HE47 |
| **48** | *H. dromedarii* | Female | *S. paucimobilis* | HE48 |
| **49** | *H. dromedarii* | Female | *S. paucimobilis* | HE49 |
| **50** | *H. dromedarii* | Female | *V. vulnificus* | HE50 |
| **51** | *H. dromedarii* | Male | *S. pseudintermedius* | HE51 |
| **52** | *H. dromedarii* | Male | *E. casseliflavus* | HE52 |
| **53** | *H. dromedarii* | Male | *S. lentus* | HE53 |
| **54** | *H. dromedarii* | Male | *S. lentus* | HE54 |
| **55** | *H. dromedarii* | Male | *Kocuria varians* | HE55 |
| **56** | *H. dromedarii* | Male | *Aerococcus viridans* | HE56 |
| **57** | *H. dromedarii* | Male | *Neis.animaloris/zoo* | HE57 |
| **58** | *H. dromedarii* | Male | *Aerococcus viridans* | HE58 |
| **59** | *H. dromedarii* | Male | *Granulicatella elegans* | HE59 |
| **60** | *H. dromedarii* | Male | *Methylobacterium spp* | HE60 |
